# Differences in the Visual Perception of Symmetric Patterns in Orangutans (*Pongo pygmaeus abelii*) and Two Human Cultural Groups: A Comparative Eye-Tracking Study

**DOI:** 10.3389/fpsyg.2016.00408

**Published:** 2016-03-30

**Authors:** Cordelia Mühlenbeck, Katja Liebal, Carla Pritsch, Thomas Jacobsen

**Affiliations:** ^1^Department of Education and Psychology, Freie Universität BerlinBerlin, Germany; ^2^Graduate School “Languages of Emotion,” Freie Universität BerlinBerlin, Germany; ^3^Experimental Psychology Unit, Helmut Schmidt University – University of the Federal Armed Forces HamburgHamburg, Germany

**Keywords:** symmetry, eye tracking, aesthetic preference, Orangutans, cultural comparison

## Abstract

Symmetric structures are of importance in relation to aesthetic preference. To investigate whether the preference for symmetric patterns is unique to humans, independent of their cultural background, we compared two human populations with distinct cultural backgrounds (Namibian hunter-gatherers and German town dwellers) with one species of non-human great apes (Orangutans) in their viewing behavior regarding symmetric and asymmetric patterns in two levels of complexity. In addition, the human participants were asked to give their aesthetic evaluation of a subset of the presented patterns. The results showed that humans of both cultural groups fixated on symmetric patterns for a longer period of time, regardless of the pattern’s complexity. On the contrary, Orangutans did not clearly differentiate between symmetric and asymmetric patterns, but were much faster in processing the presented stimuli and scanned the complete screen, while both human groups rested on the symmetric pattern after a short scanning time. The aesthetic evaluation test revealed that the fixation preference for symmetric patterns did not match with the aesthetic evaluation in the Hai//om group, whereas in the German group aesthetic evaluation was in accordance with the fixation preference in 60 percent of the cases. It can be concluded that humans prefer well-ordered structures in visual processing tasks, most likely because of a positive processing bias for symmetry, which Orangutans did not show in this task, and that, in humans, an aesthetic preference does not necessarily accompany the fixation preference.

## Introduction

Symmetry is known to be of importance with regard to visual and aesthetic preferences in a range of different contexts, such as art, skin decorations (Cárdenas and Harris, 2006), faces (Roye et al., 2008), and body shapes (Jacobsen, 2006). Various studies in experimental aesthetics have examined the perception and evaluation of symmetry (Eisenman, 1967; Day, 1968; Eisenman and Gellens, 1968; Dörner and Vehrs, 1975; Jacobsen and Höfel, 2002, 2003; Jacobsen et al., 2006; Höfel and Jacobsen, 2007; Makin et al., 2012), the importance of a balanced composition, and the degree of complexity (Berlyne, 1970, 1971, 1974) of an image. They showed that symmetries of any kind increased the preference (Eisenman, 1967; Day, 1968; Eisenman and Gellens, 1968; Jacobsen and Höfel, 2003; Höfel and Jacobsen, 2007). Jacobsen and Höfel (2002) conducted an aesthetic evaluation study and asked participants to classify symmetrical and asymmetrical graphic patterns hierarchically according to their personal aesthetic judgment. This aesthetic judgment was later also analyzed in a functional magnetic resonance imaging study (Jacobsen et al., 2006). These studies showed, as did previous studies (for review see Tyler, 1995; Treder, 2010), that a sensitivity to symmetry plays a role for human visual information processing.

The examination of the perception of symmetrical patterns is important to clarify the question of how different species structure their surrounding world visually and if they prefer preexisting ordered structures. All organisms are confronted with a variety of information in their environment and must prioritize that information which is most relevant for them. In searching for the evolutionary roots of this preference, the comparison with other closely related species could shed light on the question as to whether a fixation preference is shared with other species. In addition, a shared fixation preference could give evidence for a potentially related aesthetic preference.

Modern day aesthetics originated in the 18th century as an independent philosophical discipline, founded by A.G. Baumgarten (Riemann, 1928), as the emotionally uplifting perception of art objects (Shiner, 2003), which excluded the appreciation of natural beauty. However, as every human being is part of his or her cultural context and, hence, bound to the preferences of their cultural epoch and the associated perception expectations (Franke, 1977), it is difficult to identify the origins of aesthetic appreciation. In different societies and periods, opinions regarding the amount of information and the degree of order an artwork should contain or exhibit in order to be deemed aesthetically pleasing vary widely. According to the Russian formalists, for example, the desirable qualities of an artwork were deviation, deformation, and alienation, in contrast to the classical ordered, harmonious definition of the 18th century (Franke, 1974, 1977). Gustav Theodor Fechner’s *Vorschule der Ästhetik*, a branch of experimental psychology which evolved as a critical counterpart to classical aesthetics at the end of the 19th century (Fechner, 1897; Martin, 1906; Jacobsen, 2006), included the aesthetic appreciation of nature and tried to examine our aesthetic sensations using scientific principles, as an “aesthetic from below” (Fechner, 1897; Jacobsen, 2006).

Therefore, in exploring the biological roots of aesthetic appreciation, it is essential to use a comparative approach to experimental aesthetics. Two of the very few studies using such an approach were conducted by Morris (1962) and Rensch (1964, 1973), who examined the visual preference for symmetric, ordered structures in monkeys, great apes, and birds. Morris (1962) investigated whether great apes (six chimpanzees and one Orangutan), which were trained in drawing, were able to mark predetermined patterns and balance asymmetrical shapes. Rensch (1964, 1973) conducted a pattern recognition study with single individuals of capuchin monkeys, vervets, jackdaws, and crows, in which he analyzed how often the subjects chose a symmetrical over an unordered decorated card. In these studies Morris (1962) and Rensch (1964, 1973) found evidence for symmetric preference in these various species, but they only tested very few individuals with very special individual histories of being raised and living in close contact with humans. Therefore, it remains unclear as to whether their findings can be generalized to other monkeys and apes. From an evolutionary perspective, many different animals show preference for symmetrical features. For example, Møller (1992, 1993) showed that female barn swallows (*Hirundo rustica*) prefer males with symmetrical tail ornaments and Schlüter et al. (1998) showed that female sailfin mollies prefer males with symmetrical vertical bars. Hence, the human preference for symmetry could be adaptive, as in humans symmetrical structures in body shape and faces also have an influence on attractiveness (Thornhill and Gangestad, 1993; Grammer and Thornhill, 1994; Ramachandran and Hirstein, 1999; Rhodes, 2006). This is also supported by the cultural-comparative study of Little et al. (2007) who investigated the preference for symmetry in human faces with a group of hunter-gatherers from Tanzania. The reasons given for this preference for symmetry were, for example, that symmetric structures provide clues about the physical fitness and weakness of other organisms or that such a preference was a by-product of the way the brain processes information (Rhodes, 2006). However, the symmetry of a given trait should not be considered a signal *per se*, in the sense of having evolved only for the purpose of transmitting information. A large cohort study by Pound et al. (2014) investigated the association of fluctuating asymmetry (FA) in faces with fitness benefits or individual health, and found that the function of a preference for facial symmetry is not likely to be that it gives fitness benefits: “Facial FA was not associated with longitudinal measures of childhood health.” (Pound et al., 2014) A possible reason, Pound suggests, is that the preference for the absence of subtle asymmetry could reflect an overgeneralization from a sensitivity for negatively associated facial cues (Zebrowitz and Rhodes, 2004).

If the preference for symmetry is cross-culturally present in humans and can also be found among various species, there could be many different reasons for this. First, due to developmental and epigenetic processes across cultures, a certain behavior or preference could have evolved several times, as the importance of ontogenetic development was already pointed out by Tinbergen (1963). Second, the preference for a signal could be a by-product of cognitive recognition processes, in the sense that symmetry preferences can emerge as a by-product of generalizing or averaging out fluctuating asymmetry in stimuli and hence can be seen as a generalized learning outcome (Johnstone, 1994; Enquist and Johnstone, 1997; Swaddle et al., 2004; Pound et al., 2014). This has also been demonstrated by Ghirlanda et al. (2002) in a study with chickens. Third, the human preference for symmetry could arise from general properties of nervous systems, a shared neural substrate (Osorio, 1996; Cohen and Zaidi, 2013) rather than from face-specific adaptations. But, there is also evidence for an influence of the axis of symmetry (vertical or horizontal) on the preference for faces, which stands in contrast to the perceptual bias view and favors instead a face-specific adaptation or an experience effect (Little and Jones, 2003; Little, 2014). Little (Little, 2014) has shown that, in a comparison, preferences for symmetry were strongest for human female faces and weaker for macaque monkey faces and abstract art, which shows a domain specificity in human symmetry preferences. With regard to the domain specificity of the preference for symmetry, it also must be mentioned that Breaux et al. (2012) found no influence of symmetry on the preference for female sex swellings in chimpanzees, which shows that a preference for symmetry does not seem to occur in every domain. Hence, the question persists as to whether a preference for formal geometrical patterns could be found across cultures and species, because these stimuli are detached from biological information. Therefore, we compared humans from two cultural groups that differ drastically in regard to their social, ecological and educational background [Namibian hunter-gatherers Hai//om (or ≠Akhoe Hai//om) and German town dwellers], and compared them with one species of non-human great apes (Orangutans) from the Leipzig Zoo, Germany. The three groups also differed in regard to their exposure to built environments: in addition to receiving formal training in geometry, German pupils are much more exposed to the ordered structures–buildings and streets–as well as the enclosed living quarters of industrialized cities. The Hai//omspend most of their daily life outdoors in the northern Namibian Savannah and use allocentric notions such as north, south, east, and west to code spatial relations (Haun and Rapold, 2009), while German children spend a considerable amount of time each day inside buildings, while being accustomed to navigating in a highly structured, complex urban environment. The ≠Akhoe Hai//om have an elementary school in their village which children from the age of 5 can attend. The teaching languages are English and Khoekhoe, a language closely related to ≠Akhoe Hai//om. Children are taught general writing, reading, and mathematical skills, but without the extent of geometrical knowledge that is taught in Germany, because they have almost no opportunity to attend to secondary school. Wild Orangutans are highly arboreal and live in the canopy, immersed in a densely foliated environment with low visibility (Felton et al., 2003); in zoos, however, their environment is very different in regard to the structural complexity and availability of space. Orangutans from the Leipzig Zoo live in a less complex environment as they would in a natural rain forest, but there are nonetheless many opportunities for the apes to climb and hide in trees at various heights. Thus, we can establish that the Orangutans that were tested in the study were all familiar with the use of a three-dimensional climbing space, and that their environment differed from that of the human groups. In addition, we chose Orangutans because they represent a species with very specific evolutionary development within the range of our closest relatives, the non-human great apes. (They are the only primarily arboreal great ape; other great apes are considered semi-terrestrial. Also uniquely, Orangutans have semi-solitary social structures).

The hypotheses underlying our study are twofold. First, that symmetrical, ordered structures should be preferred, that is, fixated upon visually for a longer duration, in both Orangutans and humans with different cultural backgrounds, if the assumption is correct that symmetry offers a basis for the structuring of an individual’s surroundings in filtering out relevant information (Singer and Gray, 1995), or, at least, if the assumption is correct that symmetry often constitutes biologically relevant information. Second, we hypothesize that an additional aesthetic appreciation corresponds to this structuring behavior. It is an empirical question whether or not these two types of preferences (fixation preference and aesthetic preference) covary. Covariation was not an assumption of ours. To test the first hypothesis we used a non-invasive eye-tracking method to investigate the fixation preferences of the two human groups and compared them to the fixation preferences of the Orangutans. We measured the summed fixation duration, the number of fixations and the mean gaze point duration on the stimuli that consisted of pairs of symmetric and asymmetric graphic patterns, at two different levels of complexity. These were identical to those patterns used in the studies of Jacobsen and Höfel (2002), Höfel and Jacobsen (2003), Jacobsen et al. (2006). The benefit of this experimental method is that it reflects the visual adaptation of the three groups to their different habitats. Hunter-gatherers are much less habituated to geometrical structures than pupils from German schools, who are trained in drawing symmetrical and geometrical patterns in school lessons. If the preference for symmetry is shared with other species and represents an ability present in humans independent of their cultural background, we expect a similar fixation preference for symmetric over asymmetric patterns across these three groups. To test the second hypothesis we asked the human participants for their aesthetic judgment of the patterns to analyze whether their fixation preference matched with their aesthetic evaluation. Because, for the present study, only two cultural groups and only two species could be examined, we are aware that our results cannot lead to general evolutionary or cultural conclusions. We can only present information about the differences/similarities among these two distinct cultural groups and species.

## Materials and Methods

### Participants and Ethics Statement

The three groups for this study consisted of 27 adolescents of the Namibian hunter-gatherer group Hai//om (mean age 12.3; age range: 8–20 years; 9 males, 18 females), 25 German town dwellers (mean age 13; age range: 9–18 years; 12 males, 13 females) from two German schools in Hamburg and Berlin, and 8 Orangutans [Sumatra-Orangutans, *Pongo pygmaeus abelii*; mean age 14.5; age range: 3–32 years; 3 males, 5 females; the group consisted of 4 adult individuals (32, 24, 15, 25 years old), 1 subadult individual (9 years old) and three juveniles (two 4 years old and one 3 years old)] from the Wolfgang Köhler Primate Research Centre (WKPRC) of the Max Planck Institute for Evolutionary Anthropology (MPI-EVA) in Leipzig, Germany. The natural habitats of these three groups are very different, as described above.

Our research in Namibia was carried out in strict accordance with the ethical guidelines of the “Working Group of Indigenous Minorities in Southern Africa” (WIMSA) and received this body’s approval. A videoclip was used to present the instructions for the study to the Hai//om in their mother tongue and to ask them for their informed consent before testing, which was documented on video. Written consent could not be obtained because of illiteracy among the Hai//om. For minors the informed consent was also obtained from their parents. The participants were all recruited by and tested in a room of the school in their village. We used opportunity sampling; thus, whoever was willing to participate was tested, which explains the huge variation in age. Because of the migration behavior of the families, there was no alternative to this method.

To match the ages of the Hai//om participants, we contacted two schools in Hamburg and Berlin and tested participants with corresponding ages. Research at the German schools was conducted in accordance with the ethical recommendations of the *Deutsche Gesellschaft für Psychologie* (DGPs – German Psychological Association) and the ethical guidelines of the research institution (Freie Universität Berlin). The respective schools (their headmasters and the participating teachers) declared their interest in this study and allowed us to test pupils in different classes in order to match the age range of the Hai//om children. After the pupils agreed to participate, their parents gave their written informed consent to their participation in this study. The pupils were tested in rooms located at their schools.

Hence, informed consent was obtained for all human subjects in accordance with the Declaration of Helsinki.

Research at the WKPRC was in accordance with the recommendations of the Weatherall (2006) report on “The use of non-human primates in research.” All Orangutans were from the same group consisting of 11 individuals and lived in semi-natural indoor (230 m^2^) and outdoor (1680 m^2^) enclosures with regular feedings, daily enrichment and water *ad libitum*. All Orangutans voluntarily participated in the study, were able to stop participating at any time and were never food- or water-deprived. Research was conducted in the observation rooms (25 m^2^) and subjects were released immediately after a session was finished or if they wanted to leave. At the WKPRC, no medical, toxicological, or neurobiological research of any kind is conducted. Research was non-invasive and strictly adhered to the legal requirements in force in Germany. The study was ethically approved by an internal committee at the Max Planck Institute for Evolutionary Anthropology. The study was carried out in strict accordance with the “EAZA Minimum Standards for the Accommodation and Care of Animals in Zoos and Aquaria” (EAZA, 2008/2014), the “WAZA Ethical Guidelines for the Conduct of Research on Animals by Zoos and Aquariums” (WAZA, 2005) and the “Guidelines for the Treatment of Animals in Behavioral Research and Teaching” of the Association for the Study of Animal Behavior (ASAB, 2006).

### Apparatus

For the Orangutans and the German group, we used a screen based Tobii T 60 eye-tracker (60 Hz, Tobii Technology) with an infrared corneal reflection technique, integrated in a 17-inch TFT monitor (screen resolution 1280 pixels × 1024 pixels) and operated via an external laptop with Tobii software (version 2.1). For the Hai//om group, we used a Tobii X2 60 with the same Tobii software, also operated via an external laptop, which allowed for more flexibility in the Namibian savannah. Both eye-trackers met the same requirements for measurement accuracy and data acquisition. The Tobii X2 60 was mounted on a laptop with a 15.6 inch screen (screen resolution 1366 pixels × 768 pixels), but the stimuli had the same size as on the T 60 monitor. External eye-trackers allow the participants to move freely, which measures more natural viewing behavior.

**FIGURE 1:**
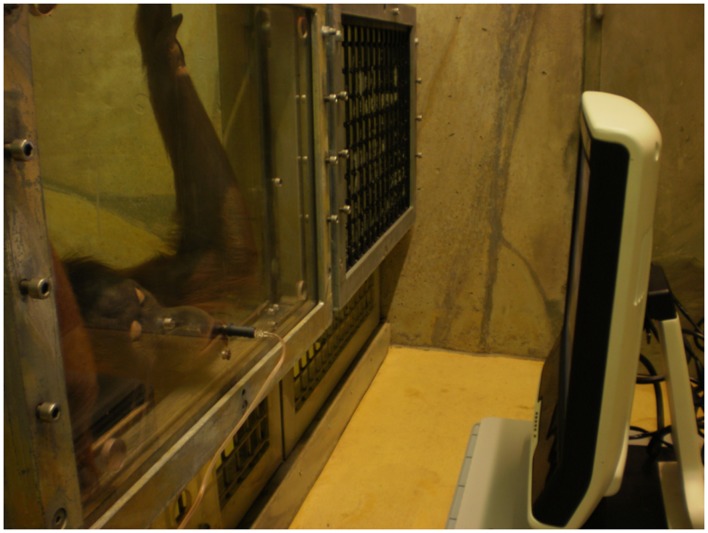
**Setting in the Orangutan observation room.** An adult female Orangutan is watching the eye-tracker while drinking juice.

**Figure 1** shows the setting at the WKPRC in the Orangutans’ observational room. They were separated from the experimenter and the eye-tracker by a plexiglass panel, through which the eye-tracker could still capture their eye movements. Through a small hole in the lower center of the panel a flexible tube was mounted to let them drink grape juice while watching. The optimal position for the eye-tracker to measure eye movements lies at a distance of between 60 and 70 cm from the subject. This distance was maintained by letting the subjects drink the juice.

For the Germans and the Hai//om, the experimental setting was placed in a room in the respective school building. All surrounding testing conditions, such as lighting conditions, were kept similar to the testing conditions of the other groups.

### Stimuli and Testing Procedure

**FIGURE 2:**
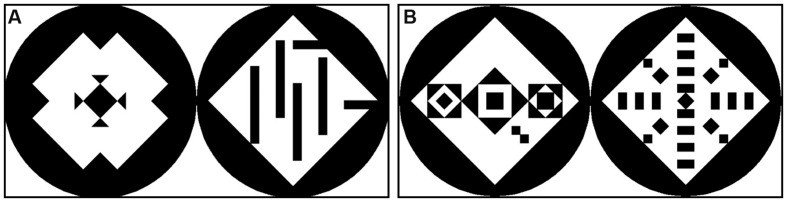
**Examples of two stimuli with simple patterns **(A)** and complex patterns **(B)**.** A number of less than 9 elements inside the square defined the patterns as simple, a number of more than 13 elements defined the patterns as complex. In the simple stimulus the symmetric pattern is left, in the complex stimulus the symmetric pattern is right. The position of the symmetric patterns was randomized.

The 60 stimuli consisted of picture pairs each comprising a symmetric and an asymmetric pattern. The stimuli set was adopted from Jacobsen and Höfel (2002). All stimuli had a size of 800 pixels × 400 pixels, were computer-generated and consisted of a black circle containing a white square, which in turn contained different formations of black triangles to create symmetric structures (see **Figure 2**). The set of 60 stimuli was divided into 30 complex and 30 simple stimuli. When the square contained more than 13 single elements, the pattern was defined as being complex; if it contained less than nine elements, it was defined as being simple. The symmetric patterns featured different types of symmetry which were chosen randomly from the original set created by Jacobsen and Höfel (2002). The different types of symmetry were: (1) only vertical symmetry (6 stimuli); (2) only horizontal symmetry (2 stimuli); (3) diagonal symmetry to both sides (14 stimuli); vertical + horizontal symmetry (23 stimuli); vertical + horizontal + diagonal to both sides symmetry (14 stimuli); diagonal symmetry only to right upper corner (1 stimulus). The asymmetric patterns featured different grades of order, randomly chosen from the original set of stimuli, always guaranteeing that all symmetry was broken. The sequence of the 60 stimuli was then arranged in eight different randomizations for the human participants. For the apes the 60 stimuli were divided into four subsets of 15 stimuli each, which were then presented in different randomizations. The position of the symmetric pattern (left or right) was balanced and randomized across the sequence to avoid an effect of reading direction and habituation. For the aesthetic preference test, the 60 stimuli were divided into 4 subsets, consisting of 15 stimuli each. One of these four subsets was presented to each participant, who was asked to evaluate the patterns according to his/her aesthetic preference, by pointing to the preferred pattern of the pair (symmetric or asymmetric).

All participants were familiar with watching a screen, but to varying degrees. While Germans were highly familiar with watching screens (TV, computer), only some of the Hai//om had occasional access to TV. However, both Hai//om and Orangutans had previously participated in screen-based eye-tracking studies. Therefore, no familiarization with screens was necessary prior to data collection. We conducted a manual two-point calibration for the Orangutans and an automated five-point calibration for the humans, where the participants had to follow the dots with their eyes, while the eye-tracker caught their corneal reflections. During calibration the calibration dots covered the whole screen to the outer edges to ensure that eye movements could be captured on the whole presented screen. To assess whether or not the calibration was successful, accuracy was checked on five points after each calibration and repeated until it showed almost the same accuracy for all participants. After successful calibration, an eye-model of each participant was saved. This method had already been successfully applied by Kano et al. (2011, 2012).

All stimuli were presented for 3 s and were separated from each other by a fixation cross shown for 0.5 s in the middle of a white screen, to avoid any influence of the fixation from one stimulus to another. For the human participants, the 60 stimuli of the eye-tracking recording were followed by the 15 stimuli of the aesthetic preference test, which together made the testing procedure last for approximately 4.5–5 min, depending on the time the participants needed for their aesthetic evaluation and the calibration. The pointing gestures of the aesthetic evaluation were documented on video for further analysis. Humans received only one session comprising the complete set of 60 stimuli. For Orangutans, only one of the four sub-tests was presented on one testing day, taking a duration of about 10–15 min including calibration and recalibration. In total they viewed the same 60 stimuli as did their human comparison groups. Measurements had to be repeated in the Orangutan group to ensure that almost all of the stimuli were fixated once. In the human groups no measurements were repeated due to higher recording rates. Double recordings of the Orangutans were later excluded from the analysis as described below. A reward consisting of a piece of fresh fruit was given to the Orangutans after the testing procedure to maintain their interest in participation. The reward was only given to them for participating in the experiments, never for their gaze behavior.

### Data Analysis

The eye-tracking fixation filter was defined to measure fixations from a duration of 100 ms, based on a radius of 50 pixels. The angular position of the eyes was recorded with a frequency of 60 Hz and matched to the coordinate system of the stimuli on the monitor. No corrections of the raw tracking data were conducted. Only for the aesthetic preference test were nine of the Hai//om participants excluded from the data analysis, because of an inability to understand the task and a software malfunction. These participants indicated to the experimenter that they had not understood the task. In the German group, one of the participants had to be excluded from the overall data analysis because of missing data due to a software malfunction. As mentioned above, measurements of the Orangutans had to be repeated due to their lower attention spans. We used the first recording of each stimulus for data analysis, when it displayed a viewing pattern of at least two gaze points. All later recordings were excluded from the analysis. In this way it was guaranteed that we only analyzed the viewing process on unknown patterns and kept the data comparable to those of the human participants, on whom no measurement repetitions had been conducted. For the statistical analysis of the participants’ viewing behavior, we created an area of interest (AOI) around the single patterns so that each stimulus consisted of two AOI, one for the symmetric and one for the asymmetric patterns. The size of the AOI was identical across all patterns and consisted of approximately 400 pixels × 400 pixels.

### Statistical Methods

Descriptive statistics (mean values) of the eye movement data were computed. For further statistical analysis, we chose to use multilevel models to comply with the hierarchical structure of our data. We had three different questions in regard to analyzing the distinctiveness of the three groups: first, the preference for symmetry or asymmetry; second, the number of gaze points on symmetry and asymmetry; third the mean fixation duration of the single gaze points. Our data structure was hierarchical in different respects: first, we had the hierarchy of individuals and groups; in addition, for questions two and three there were also several data points (several fixation points) for each trial (60 pictures). Multi-level analysis is able to take all these different influences into account (Barr, 2008)–in this case the influences of the different trials and the influences of the different individuals,–because for both the random effects have to be included into the analysis. This leads to rather relative than absolute values.

To test whether the fixation duration was influenced by the symmetry and complexity of the patterns and whether there were differences between cultural groups or humans and non-human primates, we first generated a *preference value*, which represents the relative proportion of the fixation duration on the symmetric pattern to the total fixation duration of the whole stimulus. It was calculated as follows: fixation time on symmetric pattern divided by total fixation time on stimulus, -0.5. By subtracting 0.5, the values for the preference for symmetry are set above zero and for asymmetry below zero, for better visibility. We used a general linear mixed model (GLMM, e.g., Baayen, 2008; Bolker et al., 2009), which, in the null-model, was comprised only of the *preference-value* as the dependent variable, with the fixations of all subjects in the intercept and *Subject* as random effects. Into the full-model, we included *Groups* (“Hai//om,” “Germans,” “Orangutans”), *Gender* and *Complexity* as fixed effects and *Subject* as random effects (*Model 1*). The age of the participants was inspected for being of influence, which was not found, and then excluded from the analysis, because of the incomparability of the age homogeneity of the human groups with the broad age range of the Orangutans.

We found no evidence for any left- or right-hand bias in the participants. Correlations between the fixed effects were not assumed. We checked if the assumptions of normally distributed and homogeneous residuals were fulfilled by visually inspecting a *qq*-plot and the residuals plotted against fitted values. (Both indicated no obvious deviations from these assumptions.) The model stability was examined by using the function *influence* of the R-package influence.ME (Nieuwenhuis et al., 2012) and inspecting *dfbetas*, *cook’s distance* and the *sigtest* for *Model 1*, which revealed no influential cases to exist. Variance inflation factors (VIF, e.g., Field, 2013) were applied to a standard linear model excluding the random effects and did not indicate collinearity to be an issue (R-package *car* Fox and Weisberg, 2011).

To test whether the *number of fixations* (*Model 2*) and the *mean fixation duration* of the single gaze points (*Model 3*) on the patterns was influenced by the symmetry or the complexity of the patterns and the difference in the three groups, we used again two GLMMs into which we included *Patterns* (symmetric or asymmetric), *Groups, Complexity*, and *Gender* as fixed effects, with a cross-level interaction (Baayen et al., 2008) between *Patterns* and *Groups* (to get the differences in the respective influence) and *Stimulus-Number (StimNum)* and *Subject* as cross-classified levels [cross-classified-models (Hox, 2002)], and with random slopes of *Patterns* within both. For the model of the mean duration of the gaze points (*Model 3*), we log-transformed the dependent variable to achieve better interpretability and more symmetrically distributed data. The distribution asymmetry in our data of the mean duration of the single gaze points was similar across all three groups.

Correlations between the fixed effects were not assumed. In addition, for *Models 2* and *3* no obvious deviations from the model assumptions were found (*qq*-plot and residuals against fitted values for normal distributed and homogeneous residuals). The tests for model stability [R-package influence.ME (Nieuwenhuis et al., 2012), inspecting *dfbetas*, *cook’s distance* and *sigtest* for both models] revealed that, according to classical cut-off criteria, some subjects were of influence, but according to content-based criteria they were classified as not excludable. Furthermore, VIFs did not indicate in these two models that collinearity was an issue. All models were fitted in R (R-Core-Team, 2013) using the function *lmer* of the R-package *lme4* (Bates et al., 2013). To achieve more reliable *p*-values in the full-null-model-comparison, the model was fitted using Maximum Likelihood [rather than Restricted Maximum Likelihood (Bolker et al., 2009)] and its significance was established using a likelihood ratio test (R function *anova* with argument test set to “*Chisq*”). The effect size of the variables was based on likelihood ratio tests comparing the full with respective reduced models (e.g., Barr et al., 2013).

#### Reliability

To ensure reliability for the analysis of the video data with the pointing gestures for the aesthetic preference test, a person unfamiliar with the purpose of this study coded 20% of the data. Cohen’s kappa was used to measure the degree of concordance. All measured Kappa lay between 0.93 and 1, which corresponds to a almost perfect level of agreement (Landis and Koch, 1977).

## Results

### Descriptive Mean Values

**Table 1** presents the mean values of the total fixation durations on the stimulus, for all three groups. It shows that both human groups watched for almost the entire 3 s as the stimuli were presented (Hai//om 2511 ms, the Germans 2985 ms) and that the Orangutans, probably due to their shorter attention span, watched the stimuli for 1068 ms on average. Furthermore, **Table 1** shows that the three groups differed regarding their number of fixations and their mean fixation duration on the single gaze points: the Hai//om had a longer mean duration in their single gaze points than the German participants, but with fewer gaze points than the Germans. The Orangutans had in general much shorter gaze points than the human participants. In all three groups there were differences in the fixation durations (summed mean fixation duration, see **Table 1**) and the duration of the single gaze points on symmetric patterns compared to asymmetric patterns (**Table 1**). The difference in the values between symmetric vs. asymmetric was stronger in the two human groups compared to the Orangutans, where it was less pronounced. None of the three groups showed clear differences in the number of fixations on the symmetric vs. asymmetric patterns.

**Table 1:** Descriptive mean values regarding the looking behavior of the three groups.

Descriptive mean values	Germans		Hai//om		Orangutans	
Radial distance from pattern center in pixels	55		94		129	
(Mean) number fixations on stimulus	6.7		4.6		3.1	
Total (mean) fixation duration on stimulus in ms	2985		2511		1068	
Mean fixation duration of single gaze points on whole stimulus in ms	554		680		342	

	**Symmetric**	**Asymmetric**	**Symmetric**	**Asymmetric**	**Symmetric**	**Asymmetric**

Summed (mean) fixation duration on pattern in ms	1575	1410	1351	1160	541	527
Mean fixation duration of single gaze points on pattern in ms	592	484	667	607	356	347
Number fixations on pattern	3.3	3.4	2.4	2.2	1.5	1.5


*The table shows the radial distance of the distribution of the gaze points over the patterns, calculated from the center of the pattern in pixels. Furthermore, the mean number of fixations, the mean total fixation duration and the mean fixation duration of the single gaze points are given for the whole stimulus. The summed mean fixation duration, the mean fixation duration of the single gaze points and the mean number of fixations are given for the symmetric and asymmetric patterns. Time-related information is represented in milliseconds (ms).*

### Preference Value

**FIGURE 3:**
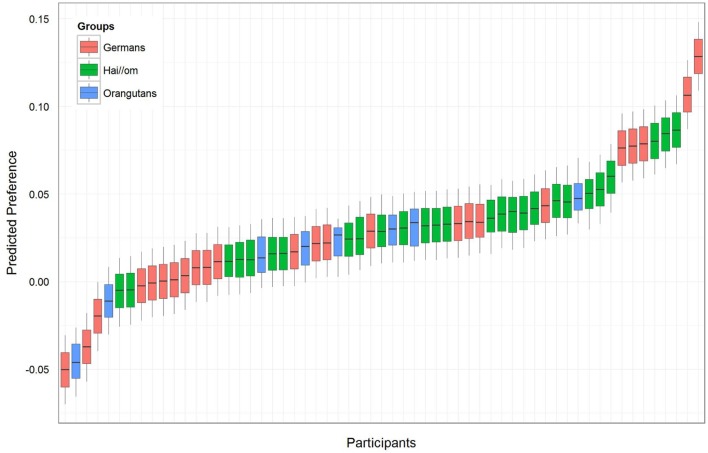
**Preference-value for symmetry for each participant.** A value above zero represents a preference for symmetric patterns and less than zero for asymmetric patterns. The different colors represent the three groups.

For the first model of the *preference-value* for the symmetric and asymmetric patterns, comparison between the full- and null-model revealed that the full model was not significant (likelihood ratio test full-model: χ^2^ = 2.471, *df* = 4, *p* = 0.650), which means that there was no effect of the factors *Groups*, *Complexity*, and *Gender*. However, since the null-model was significant (*p* < 0.001), our main hypothesis was confirmed, because it revealed an estimate of 0.029 (*SE* = 0.008) with a *t*-value of 3.63. This finding indicates a general preference for the symmetric patterns, because the preference value above zero represents a longer fixation duration on the symmetric patterns. Accordingly, we confirmed with likelihood ratio tests, comparing the full with the respective reduced models, that the factors included into the full model had no effect (*Groups*: χ^2^ = 0.815, *df* = 2, *p* = 0.665; *Complexity*: χ^2^ = 1.540, *df* = 1, *p* = 0.215; *Gender*: χ^2^ = 0.159, *df* = 1, *p* = 0.690). When the factor *Groups* showed no significant effect in the full-null-model comparison, this indicated that the differences in the estimates of the three groups are not significant, but this included no exact information about whether the preference for symmetry or asymmetry is significantly different within the three groups. For analyzing the preference within *Groups*, we ran a reduced model with only *Groups* as fixed effects to receive the respective preference values for the three groups (see **Table 2**, *Model 1*). The respective effect of each level of *Groups* is indicated by *t-*values > 0 and the respective *p*-values (R-function “*relevel*” was used to change the reference level of the factor in the intercept), which show that the effect was not significant for Orangutans, but significant for both human groups (Hai//om: estimate: 0.036, *SE*: 0.012, *t-*value: 3.078, *p* = 0.003; Germans: estimate: 0.026, *SE*: 0.012, *t-*value: 2.143, *p* = 0.037; Orangutans: estimate: 0.014, *SE*: 0.025, *t-*value: 0.560, *p* = 0.578). **Figure 3** shows the preference value for each participant. For the eight Orangutans it can be seen that the estimated values range from approximately -0.04 to 0.04. As our reduced model indicated, this is not a significant preference for symmetric patterns in the Orangutan group, though there seems to be a trend in the preference for symmetric patterns (estimate 0.014).

**Table 2:** General linear mixed model (GLMM) for fixation preference.

	Germans	Hai//om	Orangutans
**Model 1 Preference**			
	Intercept		
Estimate	0.026	0.010	-0.012
CI lower	0.002	-0.024	-0.066
CI upper	0.050	0.043	0.043
*SE*	0.012	0.017	0.027
*t-*value	2.143	0.576	-0.447


*The table shows the results for model 1 of the preference-value including *Groups* (Germans, Hai//om, Orangutans) as fixed effect. The preference-value already includes our main hypothesis, because it reflects the relative proportion of the fixation duration on the symmetric pattern, with a value above zero representing a preference for symmetry and below zero for asymmetry.*

### Number of Fixations

**FIGURE 4:**
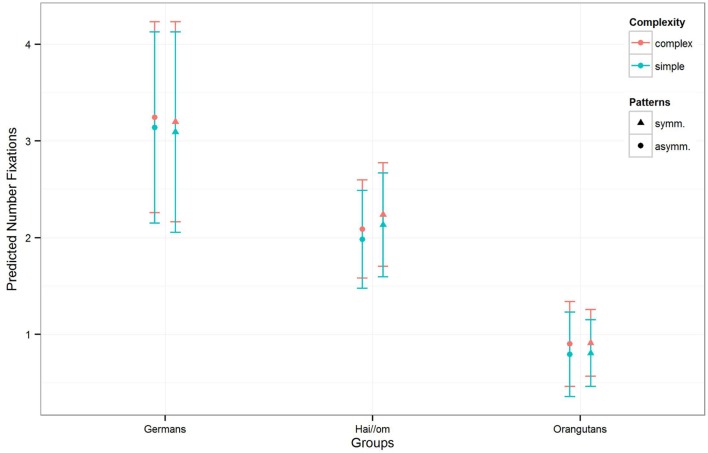
**Number of fixations for symmetry and complexity for the three groups.** The colors are indicating the complexity of the patterns, the different symbols are representing symmetry and asymmetry with the standard deviation of the estimates (of the number of fixations) in the error bars.

For the model for the *number of fixations*, the full-null-model-comparison (null-model comprising only *Gender* and the random effects; see **Table 3**, *Model 2*) was significant (χ^2^ = 172.78, *df* = 10, *p* < 0.001). In a further comparison of the full with the respective reduced models, we only found an effect for *Complexity* (χ^2^ = 4.877; *df* = 1; *p* = 0.027), but not for the other factors or their interaction (*Patterns:Groups*: χ^2^ = 3.517, *df* = 2, *p* = 0.172; *Gender*: χ^2^ = 0.337; *df* = 1; *p* = 0.562), which means that complex patterns elicited more gaze points across the three groups. The estimates and confident intervals (**Table 3**) of the interaction between *Groups* and *Patterns* show that for all groups there was no effect in the number of gaze points on symmetric or asymmetric patterns, or only a slight effect for the Hai//om group (see also **Figure 4**), which means that the participants treated symmetry and asymmetry the same way regarding the quantity of gaze points. In general, only the absolute quantity of gaze points given to the whole stimulus differed between the groups. Germans had the highest number of fixations, Orangutans the lowest, and the Hai//om lay in between.

**Table 3:** General linear mixed models for number of fixations and mean fixation duration of single gaze points.

	Germans		Hai//om		Orangutans		Complexity simple	Gender male
					
Patterns	Asymmetric	Symmetric	Asymmetric	Symmetric	Asymmetric	Symmetric		
**Model 2 number fixations**						
	Intercept							
Estimate	3.321	-0.087	-1.214	0.277	-2.370	0.083	-0.105	-0.118
CI lower	2.944	-0.322	-1.654	-0.017	-3.009	-0.348	-0.198	-0.520
CI upper	3.696	0.148	-0.775	0.572	-1.731	0.513	-0.012	0.285
*SE*	0.189	0.119	0.221	0.148	0.321	0.216	0.046	0.201
*t-*value	17.592	-0.731	-5.503	1.879	-7.390	0.382	-2.267	-0.583
**Model 3 mean fixation duration gaze points**						
Estimate	5.997	0.158	0.181	-0.089	-0.432	-0.164	0.025	0.012
CI lower	5.896	0.089	0.063	-0.178	-0.612	-0.316	-0.012	-0.093
CI upper	6.099	0.227	0.298	-0.000	-0.253	-0.011	0.062	0.118
*SE*	0.051	0.035	0.059	0.045	0.090	0.077	0.018	0.053
*t-*value	117.84	4.57	3.07	-1.99	-4.78	-2.13	1.34	0.24


*The table shows the results for the models of the number of fixations (model 2) and the mean duration of the single gaze points (model 3). The results of model 3 are calculated with the log transformed dependent variable.*

### Mean Fixation Duration Gaze Points

**FIGURE 5:**
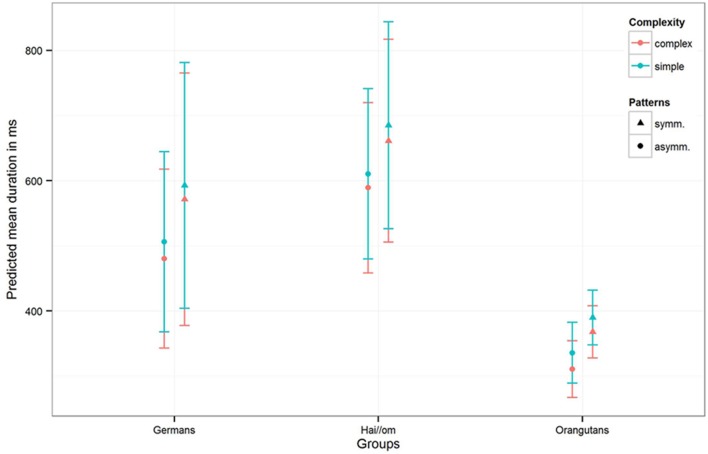
**Mean duration of the single gaze points for the three groups for symmetry and complexity.** The colors are indicating the complexity of the patterns, the different symbols are representing symmetry and asymmetry with the standard deviation of the estimates (of the mean fixation duration) in the error bars. The results are represented with not log transformed dependent variable.

The third model for the *mean fixation duration* in the single gaze points revealed for the full-null-model-comparison (comprising only *Gender* and the random effects in the null-model) that the full-model was again significant (χ^2^ = 144.11, *df* = 10, *p* < 0.001). Comparing the three groups, the estimates and confidence intervals (see **Table 3** *Model 3* and **Figure 5**) show that both human groups have similar mean gaze point durations, and, despite the Hai//om having slightly longer fixation durations than Germans, both groups have longer fixation durations than Orangutans (*Hai//om*: 600–700 ms; *Germans*: 500–600 ms; *Orangutans*: 300–400 ms, **Figure 5**). The comparison of the full with the respective reduced models revealed an effect for *Complexity* (χ^2^ = 5.345; *df* = 1; *p* = 0.021) and for the interaction of *Groups* and *Patterns* (χ^2^ = 6.261; *df* = 2; *p* = 0.044), but no effect for *Gender* (χ^2^ = 0.055; *df* = 1; *p* = 0.814). This means that the participants’ gaze points, across the three groups, were longer on symmetric patterns than on asymmetric patterns, while also longer on simple patterns than complex ones (for the estimates and confident intervals see **Table 3** *Model 3*). The results are given for the log-transformed dependent variable.

### Aesthetic Preference Test

The aesthetic preference test revealed that, in the German group, evaluation was in 61.47% (Cohen’s Kappa 1) of the cases in accordance with the fixation preference, and in the Hai//om group in 50.8% (Cohen’s Kappa 0.93) of the cases. In the German group, 20 participants pointed more often to the symmetric patterns and four to the asymmetric patterns, in aesthetically evaluating which pattern was more beautiful. In the Hai//om group, nine participants pointed more often to the symmetric patterns, eight more often to the asymmetric patterns and one had an equal number of pointing gestures (due to one case where the participant could not make a choice). As also stated in the data analysis section, for the aesthetic preference test, nine of the Hai//om participants had to be excluded from the data analysis, because of their not understanding the task and a software malfunction. In the German group, one of the participants had to be excluded from the whole data analysis because of missing data due to a software malfunction. This explains the uneven number of participants in the aesthetic preference test compared to the eye-tracking test.

### Spatial Analysis

**FIGURE 6:**
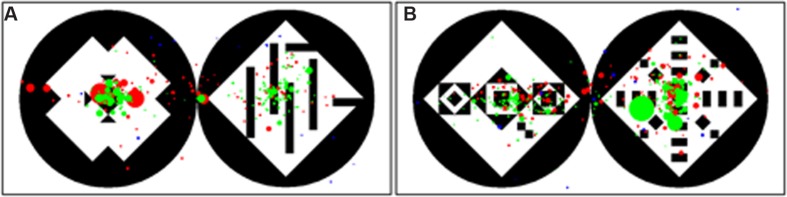
**Gaze distribution for all participants on simple **(A)** and complex **(B)** stimulus.** In the simple stimulus the left pattern is symmetric, in the complex stimulus the right pattern is symmetric. Red dots represent the gaze points of the Hai//om, green dots the gaze points of the Germans and blue the gaze points of the Orangutans. The size of the points stays for their duration – the longer the duration the larger the points.

In addition to the analysis of the temporal viewing behavior (preference in fixation duration, number fixations, mean duration of the gaze points), we also analyzed the spatial viewing behavior of the three groups. We calculated a radial distance from the center of each pattern to get information about the size of area that each participant observed. Orangutans scanned the patterns with a mean radius of 129 pixels, which corresponds to almost the whole pattern, considering that the stimuli had a height of 400 pixels. Hai//om scanned the patterns with a mean radius of 94 pixels and Germans with 55 pixels (see **Table 1** and **Figure 6**). Although the scanned radius of the Hai//om was almost twice as large compared to those of Germans, both human groups rested on the center of the symmetric patterns after an initial short scanning with smaller and faster gaze points, while Orangutans scanned the whole pattern and its surroundings (**Figure 6**).

## Discussion

We hypothesized that, first, symmetrical, ordered structures are visually preferred and that this fixation preference for symmetry should be present across cultural groups and in closely related primate species and, second, that the fixation preference for symmetrical patterns should also be accompanied by an aesthetic appreciation. The results show that both Germans and Hai//om preferred symmetric over asymmetric patterns in their fixation, suggesting that this preference might be a universal human feature, while no such preference was evident for Orangutans. Nonetheless the conclusion has to be drawn carefully since we only tested individuals from two different cultures and Orangutans with a sample size of eight individuals. The aesthetic evaluation task revealed that the fixation preference of both human groups only partly matched their aesthetic preference.

Our first analysis (full-null-model comparison revealed that the full-model was not significant) indicated that all three groups show a preference for symmetry compared to asymmetry (see estimates in **Table 2**), because the difference between the three estimates was not significant. However, when we analyzed the preference values for each of the three groups separately, only Germans and Hai//om clearly preferred symmetric over asymmetric patterns regardless of the complexity of the patterns, while there was no significant differentiation between symmetric and asymmetric patterns in the Orangutan group. Nonetheless, the Orangutans showed a trend in the preference for symmetry as revealed by the descriptive values (**Table 1**) and the model output for the fixation preference (**Table 2**). While both human groups rested on the symmetric structures after a short overall scanning of the patterns, Orangutans seemed inclined to perceive the given structures in the overall context, because they also fixated on the field surrounding the patterns.

The different fixation durations for symmetry and asymmetry and, in general, the different spatial distributions of the gazing patterns were the main differences in the comparison between species. This is consistent with the findings of one of our earlier studies comparing visual perception in Orangutans and humans (Mühlenbeck et al., 2015) and is also similar to the findings of Kano et al. (2011), who demonstrated that Orangutans and other great apes scan an image with a distribution over a large area with several short gaze points, in contrast to humans, who concentrate on small areas with larger gaze points and shorter saccades. Reasons for this different gazing behavior can only be speculated upon. In general, across primate species the visual system is very similar, which suggests similar vision abilities (Shepherd et al., 2010). From an evolutionary viewpoint, it might have been beneficial to scan a wide area very quickly for a species living in an arboreal habitat, in order to enable the discovery of unexpectedly appearing objects and animals (Kano et al., 2011). But, there are also other potential explanations for the different viewing behavior in Orangutans compared to humans, which are the pragmatically necessary differences in the experimental design, the age distribution and the sample sizes. However, except for the influence of the group size, we do not regard the differences in age distribution and experimental design as very likely explanations, because the experimental setting was kept similar and the variable *Age* did not show an effect. Thus, regarding the preference for symmetry, our findings do not support the findings of the studies by Morris (1962) and Rensch (1964, 1973), which suggested a preference for symmetric patterns in monkeys and great apes, whereby in these studies only single individuals were tested. A possible reason for the different findings could be that our sample size was larger and with the eye-tracking method a different type and amount of data can be acquired.

The number of fixations was influenced by the complexity of the patterns in all three groups. The mean gaze point duration was influenced by complexity and symmetry. Here, the complex patterns received shorter fixations in contrast to the simple patterns that were fixated upon for longer. A reason for this could be that the complex patterns contain more information to be processed, which the spectator tries to analyze by being faster.

Comparing the two human groups we found that, despite their mutual preference for symmetric patterns, they differed in regard to how they scanned the patterns. Germans fixated more often than Hai//om, while Hai//om had longer single gaze point durations (see **Figures 4** and **5**). A possible reason for this difference could be that people living in a very different ecological surrounding have a different mode of perception. Studies that have been conducted in cross-cultural comparison have shown that there are many differences in modes of human perception. For example, see the findings of Haun et al. (2006, 2011) on spatial cognition. A reason for the human preference for symmetry could be that the visual processing of the environment is simplified using given ordered structures, which could facilitate the filtering of essential information (Singer and Gray, 1995). But, however, the lack of a fixation preference in the Orangutan group poses problems for this conclusion. In the present study, only humans responded to more ordered structures, and it seems that this filtering advantage was not important for the visual perception of Orangutans. In future studies this aspect should be analyzed in a wider comparison of different species.

Regarding humans, symmetry as an organizing factor in the visual perception of the environment is well known since it was first discussed by the Gestalt school (Koffka, 1935; Kohler, 1947; Barlow and Reeves, 1979). Finding this fixation preference for symmetry in two different human cultures, living in very distinct habitats, suggests that this preference might be universally human, though more, disparate cultural groups should be tested to justify the conclusion of a shared human feature. The cultural comparative study by Little et al. (2007) had already suggested this universality for the preference for symmetric faces. Developmental studies on the recall and preference for different types of symmetric patterns (Paraskevopoulos, 1968) in 6- to 11-year-olds, and studies with 4- to 12-month-old infants (Bornstein et al., 1981; Humphrey et al., 1986) showed that the preference for symmetric structures increases during ontogeny. The 4-month-old infants processed vertical symmetry more efficiently than horizontal symmetry or asymmetry, though they did not show any preference for symmetry. While the recognition of vertical symmetry seems to be innate, since it is already present in 4-month-old infants, the preference for symmetry develops later and starts with the preference for vertical over horizontal symmetry and over asymmetry (Bornstein et al., 1981; Humphrey et al., 1986). This suggests that the aesthetic preference for symmetry is not bound to the practical benefit symmetry has for visual processing through facilitating it.

The results of our study, i.e., that both human groups, independent of their cultural background, showed a longer fixation on the symmetric patterns, but no clear matching of the aesthetic preference with the fixation preference, suggest the same conclusion, i.e., that the aesthetic preference for symmetry is not bound to the fixation preference. Only in the German group did the aesthetic appreciation of the patterns show a tendency to match with their fixation preference, which could mean that the aesthetic preference of symmetric patterns would be dependent upon the cultural background of the spectator, in contrast to the fixation preference. Accordingly, it can only be speculated about a possible aesthetic preference in the Orangutans. Since it is not possible to conduct an aesthetic evaluation task with the Orangutans, a potential aesthetic appreciation can only be inferred from their fixation behavior. The fact that in the human groups the aesthetic preference only partly matched with the fixation preference suggests that for the Orangutans, a potential aesthetic appreciation is also not very likely to match with their fixation preference, or, indeed, is even less likely, since their fixation preference was not statistically significant.

Regarding the evolutionary roots of the human fixation preference for symmetric patterns, it is not clear at what point it emerged in human evolution and whether it is unique to the human species or shared with other animals. With our research design, it was not possible to address this broader evolutionary question. Depending on the habitat, different priorities are necessary for environmental processing. A reason for humans preferring ordered structures and, contrary to Orangutans, tending to concentrate more on details in the processing of structures, could be that humans were faced with controlling and structuring new surroundings when spreading out into ever-new habitats. Symmetric structures are easier to process and thus provide a basis for the structuring of an organism’s surrounding. It has been suggested that symmetry serves as a warning system that informs us about “biologically important objects – such as predator, prey or mates” (Ramachandran and Hirstein, 1999), which are symmetrical. This cannot be supported by our findings, because it would suggest a general preference for symmetric structures in other primates and most likely also other mammals, which we, at least in these eight Orangutans, did not find.

However, Orangutans are habituated to arboreal habitats with low visibility, but the respective tested subjects are now living in a comparably poor, small and little structured captive environment. What influence this environment has on their visual perception is not clear, and it is still possible that a tendency to prefer symmetric structures also exists among Orangutans. Furthermore, the probability of representing a true effect is lower for the data of the Orangutan group, as the sample size with only eight Orangutans was smaller compared to the human groups with 24 and 27 participants. In future studies, the question as to whether symmetrical structures are preferred should be further investigated in a broader comparison of non-human great apes that are more closely related to humans. Furthermore, the aesthetic preference test should be examined in a broader cultural comparison, in order to further analyze the criteria according to which, in different cultures, patterns are regarded as beautiful. As a conclusion, our findings suggest general differences in the visual perception of symmetric patterns between humans and Orangutans. To justify the conclusion that the human fixation preference for symmetry is universally human, further investigations in a wider cross-cultural comparison would be necessary. Nevertheless, the two very different groups tested showed a clear fixation preference, but no clear aesthetic preference, for symmetry. In regard to the fixation preferences in Orangutans we can conclude that the biological traces of the preference for symmetry have to be further explored, as well as the biological traces of its appreciation.

## Author Contributions

Conceived and designed the experiment: CM, TJ, KL. Performed the experiments: CM, CP, KL. Contributed materials for the experiment: TJ. Analyzed the data: CM. Wrote the paper: CM, TJ, KL.

## Conflict of Interest Statement

The authors declare that the research was conducted in the absence of any commercial or financial relationships that could be construed as a potential conflict of interest.
